# Inhibition of Neutrophil Secretion Upon Adhesion as a Basis for the Anti-Inflammatory Effect of the Tricyclic Antidepressant Imipramine

**DOI:** 10.3389/fphar.2021.709719

**Published:** 2021-08-05

**Authors:** Svetlana I. Galkina, Ekaterina A. Golenkina, Natalia V. Fedorova, Alexander L. Ksenofontov, Marina V. Serebryakova, Evgenii A. Arifulin, Vladimir I. Stadnichuk, Ludmila A. Baratova, Galina F. Sud’ina

**Affiliations:** ^1^ A.N. Belozersky Institute of Physico-Chemical Biology, M.V. Lomonosov Moscow State University, Moscow, Russia; ^2^ Physical Department of M.V. Lomonosov Moscow State University, Moscow, Russia

**Keywords:** imipramine, neutrophil, adhesion, secretion, NGAL, MMP-9, hydroxylysine, lysyl hydroxylase

## Abstract

Recent studies demonstrate the involvement of inflammatory processes in the development of depression and the anti-inflammatory effects of antidepressants. Infiltration and adhesion of neutrophils to nerve tissues and their aggressive secretion are considered as possible causes of inflammatory processes in depression. We studied the effect of the antidepressant imipramine on the adhesion and accompanied secretion of neutrophils under control conditions and in the presence of lipopolysaccharides (LPS). As a model of integrin-dependent neutrophil infiltration into tissues, we used integrin-dependent adhesion of neutrophils to the fibronectin-coated substrate. Imipramine inhibited neutrophil adhesion and concomitant secretion of proteins, including matrix metalloproteinase 9 (MMP-9) and neutrophil gelatinase-associated lipocalin (NGAL), which modify the extracellular matrix and basement membranes required for cell migration. Imipramine also significantly and selectively blocked the release of the free amino acid hydroxylysine, a product of lysyl hydroxylase, an enzyme that affects the organization of the extracellular matrix by modifying collagen lysine residues. In contrast, imipramine enhanced the release of ROS by neutrophils during adhesion to fibronectin and stimulated apoptosis. The anti-inflammatory effect of imipramine may be associated with the suppression of neutrophil infiltration and their adhesion to nerve tissues by inhibiting the secretion of neutrophils, which provides these processes.

## Introduction

Imipramine, the oldest tricyclic antidepressant, is used to treat chronic psychiatric disorders, including major depressive disorder (MDD) and related diseases (Wille et al., 2008). Although antidepressants have been used in therapy for over 50 years, the mechanism of action of these drugs remains unclear. Recently, new pharmacological effects of antidepressants have been discovered, including anti-inflammatory effects. Stimuli such as inflammation, chronic stress and infection can trigger the activation of microglia, the brain’s immune cells, to release pro-inflammatory cytokines that may lead to MDD and neurodegeneration (Kopschina Feltes et al., 2017). The neuroprotective effect of imipramine and other antidepressants may be associated, at least in part, with the inhibition of the inflammatory response of glial cells (Hashioka, 2011; Obuchowicz et al., 2014). Other immune cells that can play a key role in inflammation associated with chronic psychiatric disorders are neutrophils.

A characteristic property of neutrophils is the ability to migrate from the bloodstream and penetrate into the tissues of the body during infection or certain metabolic disorders, such as reperfusion after ischemia or diabetes (Patel, 2009; Schofield et al., 2013). Once in the tissues of the body, aggressive products of neutrophil secretion, designed to destroy pathogenic microbes, aggravate inflammatory processes in the vessels and surrounding tissues. Aggressive secretion of neutrophils includes bactericidal enzymes and pore-forming peptides, which are localized in intracellular granules of three types, and reactive oxygen species (ROS) formed by the NADPH oxidase complex, which collects on the membranes of activated neutrophils (Segal, 2005; Winterbourn and Kettle, 2013).

Infiltration of neutrophils in the CNS, their adhesion and concomitant secretion can contribute to the development of inflammation and numerous neurological and neurodegenerative diseases, including MDD (Prinz and Priller, 2017; Kanashiro et al., 2020). Repetitive social defeat stress induces the mobilization of neutrophils in mice, which may contribute to the development of mental illness (Ishikawa et al., 2020). Genetic analysis revealed that MDD is associated with increased expression of innate immune and neutrophil-related genes in peripheral blood (Wittenberg et al., 2020). Depression cases, compared with controls, had significantly increased immune cell counts, especially neutrophils and monocytes (Maes et al., 1992; Lynall et al., 2020). Increased neutrophil/lymphocyte ratio was observed in patients with depression or other psychiatric diagnoses (Mazza et al., 2018; Brinn and Stone, 2020). Elevated neutrophil-to-lymphocyte ratio was shown to predict depression after intracerebral hemorrhage (Gong et al., 2020). Neutrophils are the first cells which are recruited into the brain within minutes after stroke. They increase ischemic injury and impair behavior in stroke (Neumann et al., 2015; Ruhnau et al., 2017; Ritzel et al., 2018).

Bacterial lipopolysaccharides (LPS, endotoxins) of the outer membrane of gram-negative bacteria can play an important role in the regulation of neutrophil activity and infiltration (Alexander and Rietschel, 2001). The source of LPS can be bacteria that have entered the body from the environment, or bacteria in the gastrointestinal tract. The interaction between the gastrointestinal tract and brain function has recently become a topic of growing interest in psychiatric research (Carlessi et al., 2019). Immune activation associated with intestinal LPS has been observed in major depression and other mental illnesses (Maes et al., 2008; Rudzki and Szulc, 2018).

In this work, we studied how imipramine affects the activity of neutrophils under control conditions and in the presence of LPS. We have used neutrophil adhesion to fibronectin as a model for integrin-dependent adhesion (Galkina et al., 2019; Galkina et al., 2021). Our previous data revealed that neutrophils adhered well and spread on fibronectin-coated substrates. Concomitant secretion included: a component of primary granules myeloperoxidase, secondary granule components, albumin and some cytosolic proteins (Galkina et al., 2012; Galkina et al., 2017). The morphology of neutrophils attached to fibronectin in the presence of LPS was practically similar, but the secretion of neutrophils was enriched with tertiary granular components such as matrix metalloproteinases (MMPs) and primary granular components such as cathepsin G and defensins (Galkina et al., 2017). MMPs play an important role in neutrophil migration and recruitment into tissue (Dejonckheere et al., 2011). Aggressive bactericides cathepsin G and defensins, once in the environment, can initiate inflammatory processes (Eipper et al., 2016).

Amino acid analysis showed that adhesion to fibronectin sharply and selectively stimulates the secretion of hydroxylysine by neutrophils (Galkina et al., 2019; Galkina et al., 2021) but does not affect the release of other amino acids. Hydroxylysine is a lysine metabolite produced by lysyl hydroxylase (LH 1-3 or procollagen lysine, 2-oxoglutarate-5-dioxygenase, PLOD 1-3) that modifies collagen lysine residues in the rough endoplasmic reticulum and plays a key role in collagen deposition and extracellular matrix organization (Risteli et al., 2009). In tumor cells, LH is also secreted outside and modifies proteins in the extracellular environment (Salo et al., 2006; Wang et al., 2012; Chen et al., 2016). PLOD3 and PLOD2 are overexpressed and secreted by cells of lung cancer (Baek et al., 2018; Baek et al., 2019) glioma (Tsai et al., 2018), glioblastoma (Verano-Braga et al., 2018) and pancreatic duct adenocarcinoma (Schiarea et al., 2010). These enzymes promote cancer development and metastasis and are considered potential targets for cancer treatment.

To assess whether the neuroprotective effect of imipramine is associated with inhibition of neutrophil infiltration and neutrophil-induced inflammation, we examined the effect of imipramine on neutrophil adhesion to a fibronectin-coated substrate and concomitant secretion of proteins, free amino acids and reactive oxygen species, as well as on apoptosis under control conditions or upon stimulation with LPS. We used scanning and transmission electron microscopy to study neutrophil morphology, electrophoretic separation and mass spectrometric identification of secreted proteins, amino acid analysis to study the composition of free amino acid secretion, and flow cytometry to study apoptosis.

## Materials and Methods

### Materials

Bicarbonate-free Hank’s solution, Ca2^+^-free Dulbecco PBS, imipramine, LPS (lipopolysaccharide from *Salmonella enterica* serovar Typhimurium) and E64 were obtained from Sigma (Steinheim, Germany). Ficoll-Paque was obtained from Pharmacia (Uppsala, Sweden). Fibronectin was from Calbiochem (La Jolla, United States). Coomassie Brilliant Blue G-250 was obtained from Serva, PMSF from MP Biomedical, trypan blue from Fluka AG, glutaraldehyde from Ted Pella. Trypsin was from Promega, carboxy-H_2_DCF-DA from Molecular probe, United States. Analytical chromatography conditions: eluent MCI Buffer L-8800-PH-1–4 and ninhydrin coloring solution kit for Hitachi 29970501 (Wako Chemicals GmbH, United States).

### Neutrophil Isolation

Neutrophils were isolated from the blood of healthy volunteers who had not taken medication for 2 weeks. All donors gave their informed consent. The study was approved by the Bioethics Commission of M.V. Lomonosov Moscow State University, application # 6-h version 3, approved during the Bioethics Commission meeting # 131-days held on May 31, 2021.

Erythrocytes were precipitated in the presence of 3% T-500 dextran at room temperature. Neutrophils were isolated from plasma by centrifugation through Ficoll-Paque at a density of 1.077 g/ml, followed by hypotonic lysis of the remaining erythrocytes in buffer (114 mM NH_4_Cl, 7.5 mM KHCO_3_, 100 μM EDTA) and washing in PBS. Before experiments, neutrophils were stored in Dulbecco PBS containing 1 mg/ml glucose (no CaCl_2_). Neutrophils accounted for 96–97% of the total number of cells in the preparation. The viability of neutrophils was determined by staining with trypan blue dye, which stains dead cells but does not penetrate viable cells. Neutrophils were incubated with 0.5 mM trypan blue in Hanks solution for 15 min at 37°C, washed, and the number of dead cells was counted. The percentage of dead cells did not exceed 1–2% of the total number of counted cells (3,000 cells per group).

### Adhesion of Neutrophils to Culture Plates Coated With Fibronectin

Cellstar six-well culture plates (Frichenhausen, Germany) were coated with fibronectin for 2 h incubation with fibronectin (5 μg/ml) in Hank’s solution at room temperature and washed. Neutrophils were attached to fibronectin-coated wells (3 × 10^6^ cells in 1.3 ml/well) for 20 min incubation in Hank’s solution containing 10 mM HEPES (pH 7.35) at 37°C. LPS and imipramine were added to the cells prior to incubation. After incubation, samples of the extracellular medium were taken and mixed with inhibitors of metalloproteinase, serine and cysteine proteinases, and myeloperoxidase (EDTA, 5 mM; PMSF, 200 μM; E64, 10 μM; and sodium azide, 0.025%, respectively). Neutrophils remaining in the extracellular medium were removed by centrifugation (5 min at 400 x g at room temperature). Extracellular medium samples from three identical wells were pooled for amino acid analysis. To determine the protein content in the secretion of neutrophils, samples from six identical wells were combined.

### Quantification of Neutrophil Adhesion

Neutrophils (2 × 10^5^ cells/probe) in HBSS/HEPES, supplemented or not with imipramine, were incubated in fibronectin-coated 96-well plates for 30 min at 37°C in 5% CO_2_. Supernatants were then carefully removed followed by double washing with warm PBS to remove free-floating or weakly attached cells. Quantification of adhesion was carried out according to the method described by Ngo and coauthors (Ngo and Lenhoff, 1980; Sud’ina et al., 2001). Briefly, hydrogen peroxide (4 mM final concentration) in permeabilizing buffer (67 mM Na_2_HPO_4_, 35 mM citric acid, 0.1% Triton X-100) supplemented with 5.5 mM o-Pphenylenediamine dihydrochloride (OPD) was added to substrate-bound neutrophils for 5 min. MPO-catalyzed oxidation of OPD by H_2_O_2_ leading to the formation of colored product 2, 3-diaminophenazine was stopped by adding of 1M H_2_SO_4._ The absorption was measured at a wavelength of 490 nm and compared with the calibration values.

### Isolation of Proteins and Separation by Electrophoresis in Polyacrylamide Gel with Sodium Dodecyl Sulfate.

Proteins from samples of the extracellular medium were extracted with an equal volume of chloroform-methanol (2: 1, v/v), as previously published (Galkina et al., 2012). The chloroform phase was separated by centrifugation for 20 min at 11,000 x g, collected and, after evaporation of the solvent, subjected to electrophoresis. Proteins were separated by one-dimensional electrophoresis in the presence of sodium dodecyl sulfate under non-reducing conditions on a 15% polyacrylamide gel in a Mini-PROTEAN 3 cell (Bio-Rad). Aliquots of the samples were boiled for 3 min in lysis buffer (Tris-HCl 30 mM, pH 6.8; SDS 1%; urea 3 M; glycerol 10%; bromophenol blue 0.02%) before electrophoresis. Protein bands were stained with 0.22% Coomassie brilliant blue G-250.

### Protein Identification by Mass Spectrometry

Matrix assisted laser desorption ionization mass spectrometry (MALDI-MS) analysis of proteins was performed with a MALDI-ToF-ToF mass spectrometer Ultraflextreme (Bruker, Karlsruhe, Germany) as previously described (Galkina et al., 2021). Protein hydrolysis with trypsin was performed directly in the gel. Gel pieces were excised from each protein band, washed, dehydrated, air dried, and subjected to trypsin digestion in the gel. The peptides resulting from hydrolysis were extracted with 0.5% trifluoroacetic acid. Aliquots were taken from each sample and mixed on a steel target with 2,5-dihydroxybenzoic acid (30 mg/ml in 30% acetonitrile and 0.5% trifluoroacetic acid), dried and subjected to mass spectrometric analysis. The [MH]^+^ molecular ions were measured in reflector mode; the accuracy of mass peak measurement was within 30 ppm. Identification of proteins was carried out by a peptide fingerprint search using Mascot software 2.5.01 (http://www.matrixscience.com, accessed on January 3, 2021), SwissProt database through the mammalian proteins. When the score was >68, protein matches were considered significant (*p* < 0.05).

### Sample Preparation and Amino Acid Analysis

Samples of the extracellular medium, which were combined from three identical wells, were concentrated using a Centrivap Concentrator Labconco (United States), then the proteins were precipitated with sulfosalicylic acid (4.4%). The sediments were removed by centrifugation for 30 min at 18,000 x g. Supernatants were centrifuged through Vivaspin 500 Membrane 3000 PES MWCO membrane ultrafilters (Sartorius, Germany) and subjected to amino acid analysis.

The amino acid analysis was conducted on an L-8800 amino acid analyzer (Hitachi, Tokyo, Japan) in the standard mode according to the manufacturer’s user manual (Hitachi High-Technologies Corporation, Japan, 1998) as described previously (Galkina et al., 2019). The prepared samples were separated on a 2622SC-PH ion-exchange column (Hitachi, Ltd., P/N 855-3,508, 4.6*80 mm) by step gradient of four sodium-acetate buffers at an elution rate 0.4 ml/min at 57°C. The stained products were detected by measuring the absorbance at 570 nm for all amino acids except proline and at 440 nm for proline. MultiChrom for Windows software (Ampersand Ltd., Moscow, Russia) was used for processing the chromatographic data.

### Scanning Electron Microscopy

For coating with fibronectin the cover slips were incubated in a buffer containing 5 μg/ml fibronectin for 2 h at room temperature and washed. Neutrophils were attached to the fibronectin-coated cover slips (3 × 10^6^ cells in 2 ml per well) during 20 min incubation in a Hanks solution containing 10 mM HEPES (pH 7.35) at 37°C. LPS (10 μg/ml) and imipramine (100 µM) were added to the cells before incubation. After incubation, attached neutrophils were fixed in 2.5% glutaraldehyde in Hanks buffer without Ca^2+^ or Mg^2+^ ions, but containing 5 mM EDTA and 0.5 mM phenylmethylsulfonyl fluoride (PMSF), metalloproteinase and serine proteases inhibitors, and 10 mM HEPES at pH 7.3. In addition, the cells were fixed with a 1% solution of osmium tetroxide in 0.1 M sodium cacodylate containing 0.1 M sucrose at pH 7.3. Then the cells were dehydrated in a series of acetones (10–100%) and dried in a Balzer apparatus at the critical point with liquid CO_2_ as a transition liquid. Samples coated with gold/palladium sputtering were then examined at 15 KV with a scanning electron microscope Camscan S-2. The area occupied by the cells on the substrate was measured quantitatively using an ImageJ-win64 software in scanning electron microscopy images.

### Transmission Electron Microscopy

Neutrophils attached to fibronectin-coated coverslips under control conditions or in the presence of imipramine were fixed in the same way as for scanning electron microscopy. Fixed samples were dehydrated in the usual way (70% ethanol containing 2% uranyl acetate), embedded in Epon 812 (Fluka), cut into ultrathin sections with a Reichert Ultra Cut III and stained with lead citrate. The internal morphology of the cell was examined using a JEM-1400 transmission electron microscope.

### Actin Cytoskeleton Staining

Neutrophils attached to coverslips under control conditions or in the presence of imipramine were fixed in 4% paraformaldehyde in HEPES buffer free of Ca^2+^ and Mg^2+^ containing 5 mM EDTA (pH 7.3). Then the cells were treated with 0.1% Triton X-100 solution for 10 min to increase the permeability. FITC phalloidin was used to stain actin. Phase contrast and fluorescence images of neutrophils were observed using a Zeiss Axiovert 200M microscope.

### Monitoring of ROS Formation

The formation of intracellular ROS was monitored by measuring the green fluorescence of the oxidation product (DCF) of dichlorodihydrofluorescein diacetate (H_2_DCF-DA, Molecular probe, United States). Human neutrophils were incubated with 5 μM carboxy-H_2_DCF-DA for 60 min at room temperature and washed with PBS according to the manufacturer’s protocol. The cells were then plated onto fibronectin-coated 96-well plates (1 × 10^6^/ml HBSS/HEPES) during incubation according to the experimental protocol at 37°C in 5% CO_2_ under control conditions and in the presence of 10 or 100 µM imipramine. DCF fluorescence signals (excitation 485 nm, emission 538 nm) were monitored at 10 min intervals on ClarioStar fluorescence microplate reader (BMG Labtech, Ortenberg, Germany).

### Apoptosis Assessment

We studied phosphatidylserine externalization and membrane integrity of neutrophils exposed to imipramine using simultaneous staining with Annexin V-Alexa Fluor 488 and nonvital dye propidium iodide (AnnV/PI) followed by flow cytometry. Neutrophils were suspended at a density of 1 × 10^6^ cells/mL in HBSS containing 10 mM HEPES under control conditions or in the presence of 10 or 100 μM imipramine and incubated for 4 h at 37°C in a 5% CO_2_ incubator. After incubation, cells were sedimented by centrifugation at 270 × g and resuspended in Annexin V-Alexa Fluor 488 commercial solution (Merck, Germany) according to the manufacturer’s instructions. After 10 min on ice, propidium iodide (Merck, Germany) solution (10 μg/ml HBSS/HEPES) was added for 5 min. The samples were analyzed on CytoFLEX flow cytometer (Beckman Coulter, Krefeld, Germany) using CytExpert 2.0 software. Fluorescence was detected by photomultipliers at 525 nm (AnnV) and 620 nm (PI). Leukocyte subpopulations were plotted as a dot plot and gated according to size and granularity. 20,000 data events were collected for each acquisition.

For DNA fragmentation assessment, PMNLs were suspended at a density of 1 × 10^6^ cells/mL in RPMI 1640 medium (10% fetal bovine serum) and incubated for 18 h at 37°C in a 5% CO_2_ incubator. Then cells were harvested, supplemented with ice-cold 0.05% BSA in PBS, collected by centrifugation and permeabilized in cold hypotonic PI solution (20 μg/ml PI, 0.2 mg/ml RNase in 0.1% Triton X-100 in 0.1% sodium citrate). The tubes were placed at 4°C in the dark for 10–15 min before flow cytometric analysis using CytoFLEX flow cytometer (Beckman Coulter, Krefeld, Germany) with excitation and emission wavelengths of 480 ± 10 and 585 ± 20 nm, respectively.

### Statistics

Each experiment to determine the amino acid or protein composition of neutrophil secretion was performed at least three times using blood from different donors. Experiments on electron microscopic determination of the morphology of neutrophils were repeated three times using the blood of different donors. Results are represented as mean ± SEM. The statistical significance was estimated using GraphPadPrism7 software.

## Results

### Effect of Imipramine on the Morphology and Actin Cytoskeleton of Neutrophils Attached to Fibronectin.

**FIGURE 1:**
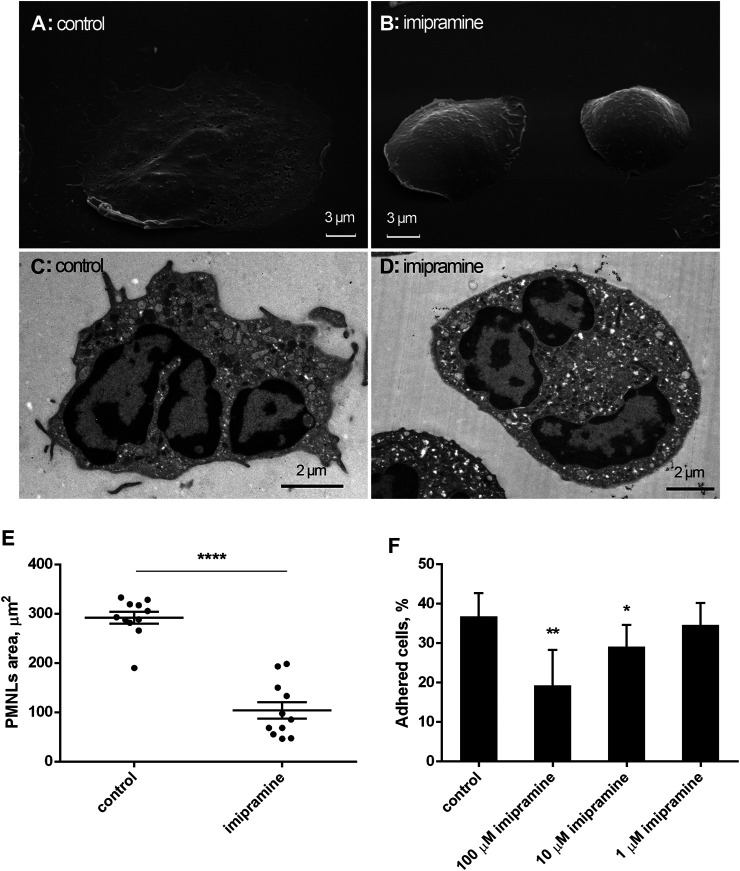
Effect of imipramine on the morphology of neutrophils attached to a fibronectin-coated substrate. Scanning **(A, B)** and transmission **(C, D)** electron microscopy images of human neutrophils that were attached to fibronectin-coated substrates for 20 min under control conditions **(A, C)** or in the presence of 100 μM imipramine **(B, D)**. Images are typical images observed in three independent experiments. The effect of imipramine on the area occupied by attached neutrophils on the substrate **(E)**. Neutrophils were attached to the substrate during 20 min under control conditions or in the presence of 100 µM imipramine. The cell area was measured in images of cells obtained by scanning electron microscopy using ImageJ-win64 software. ****—*p* < 0.0001 as indicated by unpaired *t* test (*n* = 11) The effect of imipramine on the proportion of neutrophils firmly attached to the substrate as the percentages of total number of cells **(F)**. Neutrophils were attached to the substrate during 20 min under control conditions or in the presence of 1, 10 or 100 µM imipramine. After double washing with warm PBS to remove free-floating or weakly attached cells the number of adherent cells was estimated by chromogenic assay of myeloperoxidase-coupled o-phenylenediamine dihydrochloride oxidation. *—*p* < 0.05; **—*p* < 0.01, compared with control as indicated by ordinary one-way ANOVA (*n* = 3).

We compared the morphology of neutrophils that were attached to the extracellular matrix protein fibronectin under control conditions and in the presence of imipramine using scanning and transmission electron microscopy. Neutrophils adhered and spread on fibronectin under control conditions (Figures 1A,C), while cell adhesion was partially inhibited in the presence of imipramine (Figures 1B,D). Imipramine reduced the area occupied by neutrophils on the substrate, which was measured in images of control and imipramine-treated cells obtained by scanning electron microscopy using ImageJ-win64 software (Figure 1E). The average area of the control cells was 232 μm^2^ more than two times overcame the area of cells attached in the presence of imipramine 104 μm^2^ (Figure 1E). In contrast to the well-attached control cells, the edges of imipramine-treated neutrophils were attached to the substrate only in part indicating poor attachment. The number of firmly attached control and imipramine treated neutrophils were compared after double washing with warm PBS to remove free-floating or weakly attached cells. Our data indicated that 10–100 μM imipramine statistically significant reduced cell attachment (Figure 1F). Internal morphology of neutrophils, examined by transmission electron microscopy, did not reveal specific differences in the intracellular structures of control neutrophils and neutrophils treated with imipramine (Figures 1C,D).

**FIGURE 2:**
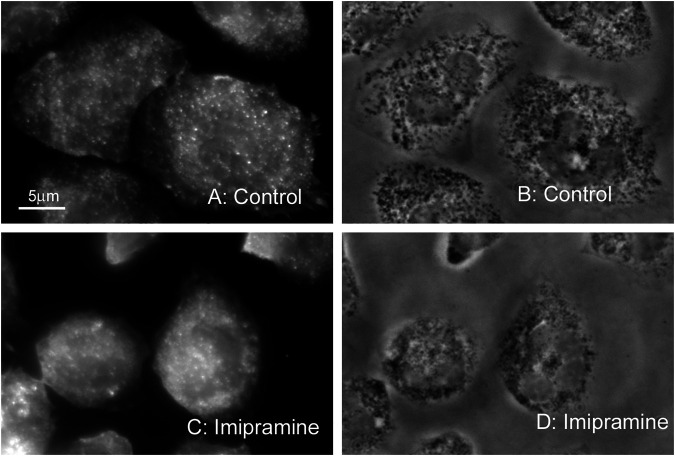
Effect of imipramine on the actin cytoskeleton of neutrophils attached to a fibronectin-coated substrate. Fluorescent **(A, C)** and phase contrast **(B, D)** images of neutrophils attached to fibronectin-coated substrates under control conditions **(A, B)** or in the presence of 100 μM imipramine **(C, D)** for 20 min at 37°C. Neutrophils were stained for actin with phalloidin FITC. Images are typical images observed in three independent experiments.

We also compared the organization of actin cytoskeleton in neutrophils that adhered to fibronectin under control conditions and in the presence of imipramine using fluorescent microscopy technique. The actin cytoskeleton undergoes depolymerization during the adhesion of neutrophils to fibronectin. The concentration of filamentous actin decreases, while monomeric actin increases during the first minutes of adhesion and then partial remodeling of the actin filaments occurs (Ginis et al., 1992; Wang et al., 1993). In our experiments, fluorescent actin staining showed that neutrophils placed on fibronectin under control conditions had diffuse actin staining of the entire cell with small actin filaments. There was no discernible difference in actin cytoskeleton between neutrophils that were attached to fibronectin under control conditions or in the presence of imipramine (Figure 2).

### Dose-Dependent Pro-Oxidant Action of Imipramine

**FIGURE 3:**
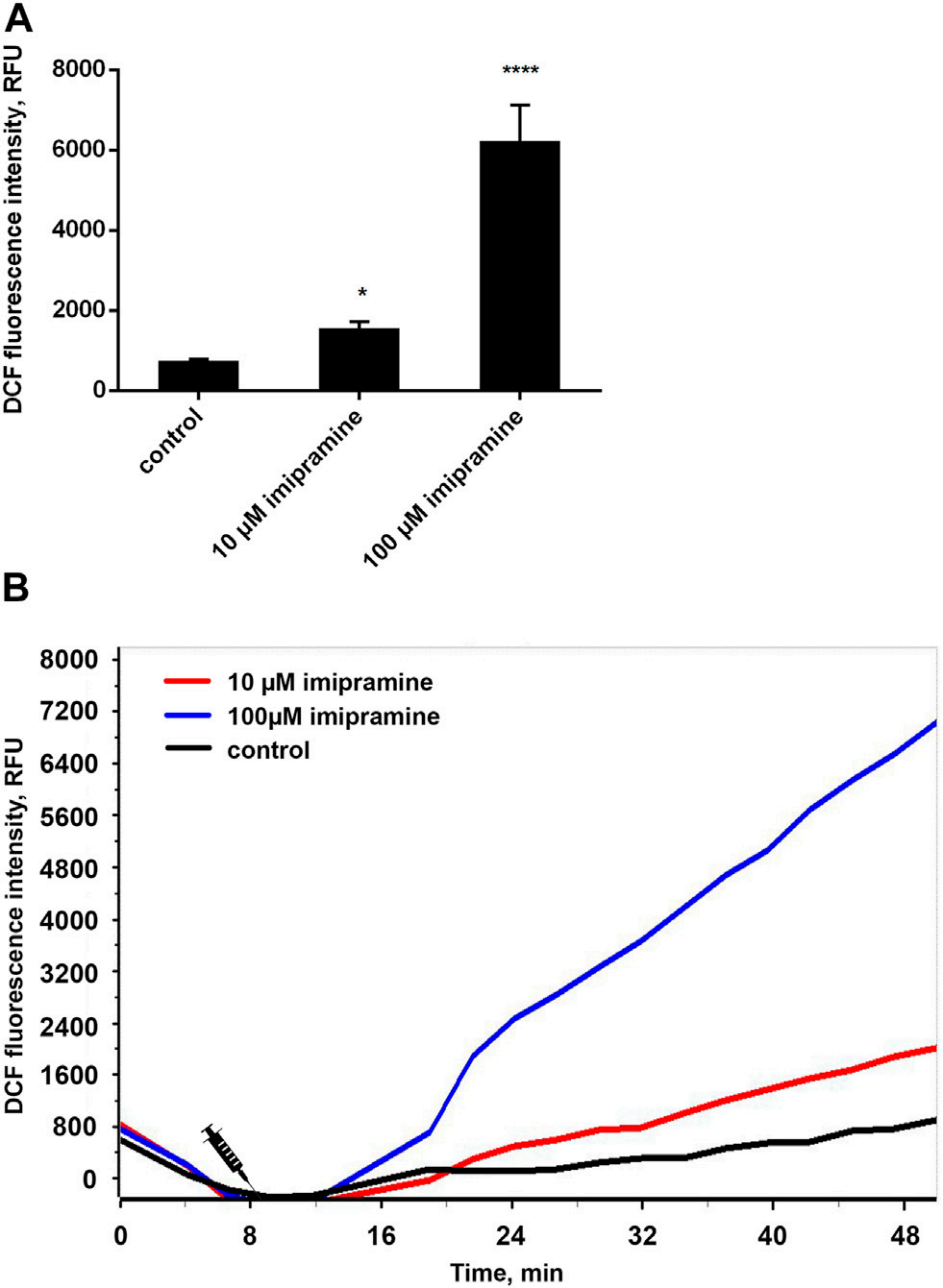
Imipramine stimulated the ROS production by neutrophils during adhesion to fibronectin. **(A)** H_2_DCF-DA-stained neutrophils were incubated in fibronectin-coated 96-well plates for 60 min at 37°C in 5% CO_2_ under control conditions and in the presence of 10 µM or 100 µM imipramine. Green DCF fluorescence was measured for every 10 min throughout the entire incubation period. Values represent the means ± SEM of DCF fluorescence intensity (relative units) 30 min after adding stimuli from three independent experiments.^*^ *p* < 0.05, ^****^ *p* < 0.0001 compared with control as indicated by ordinary one-way ANOVA. **(B)** Exemplary kinetic curves show an increase of DCF fluorescence, meaning an increase of intracellular ROS in neutrophils.

We studied the effect of imipramine on intracellular ROS production by neutrophils during adhesion to fibronectin by measuring the green fluorescence of DCF, an oxidized product of H2DCF-DA. The adhesion of neutrophils to the fibronectin-coated substrate occurs via integrin β-1 and β-2. The binding of β-2 integrin leads to the spreading of neutrophils along the substrate, the production of ROS and the outflow of chloride ions (Menegazzi et al., 1999). Assembly of the NADPH oxidase complex also occurs in response to the binding of β-1 integrin to the high-affinity binding site on fibronectin and following assembly and activation of focal adhesion complexes (Umanskiy et al., 2003). Our data demonstrated that adhesion to fibronectin itself initiated the ROS formation by neutrophils (Figure 3). Imipramine in the concentration range from 10 to 100 μM did not suppress, but significantly stimulated the production of ROS by neutrophils (Figure 3).

### Apoptogenic Effect of Imipramine

**FIGURE 4:**
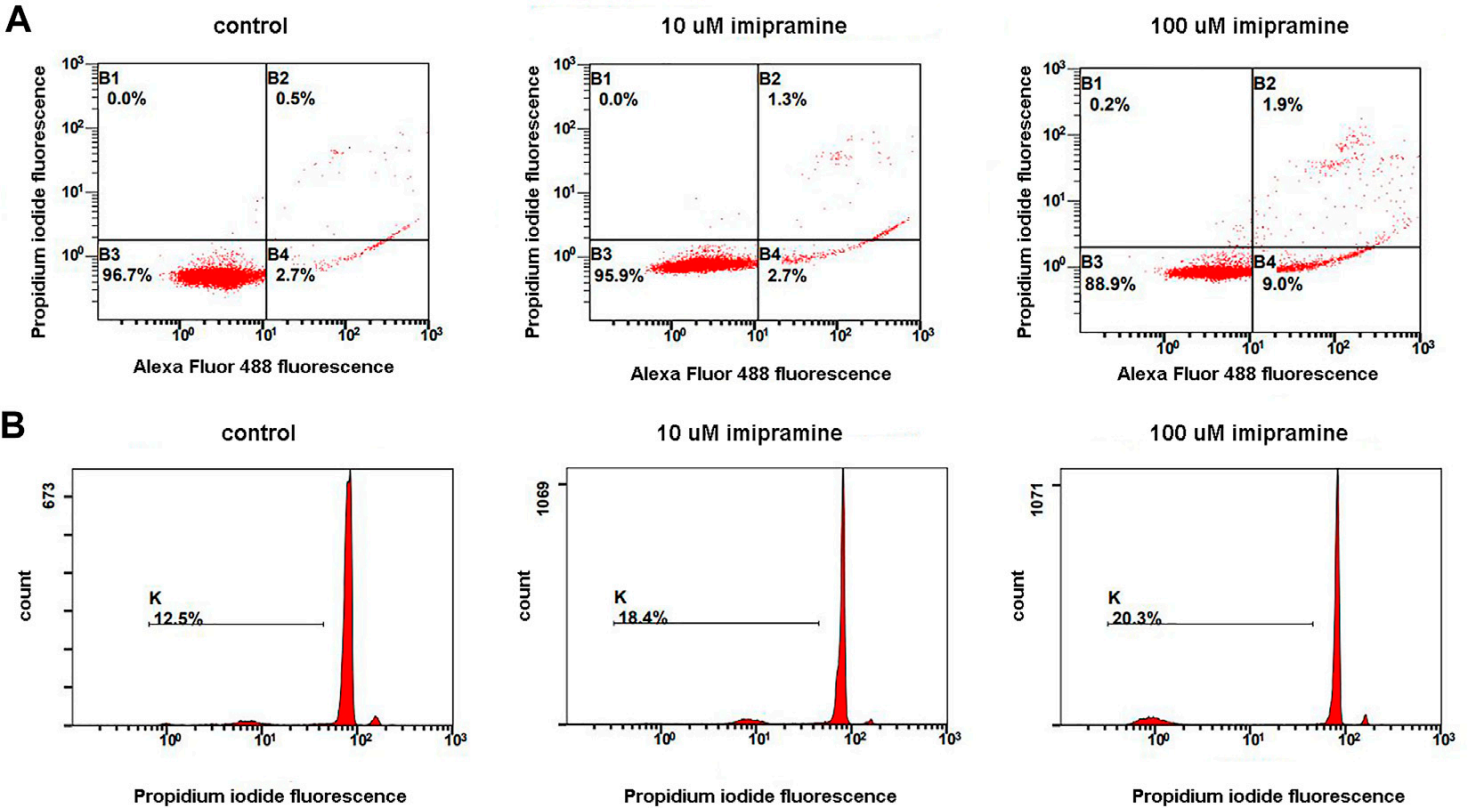
A dose-dependent apoptogenic effect of imipramine on neutrophils were cultured in RPMI-medium at 37°C, 5% CO_2_ under control conditions or in the presence of 10 or 100 µM imipramine. Early apoptosis (4 h incubation) was assessed by the severity of membrane changes using double AnnexinV/PI-labeling. Late apoptosis (18 h) was judged by DNA fragmentation quantifying using PI-labeling of permeabilized cells **(B)**. **(A)** Representative dot plots indicative of apoptosis by phosphatidylserine externalization, as well as the proportions of viable (region B3), early apoptotic (region B4), and late apoptotic and necrotic cells (regions B2 and B1), are indicated for the control and imipramine-treated neutrophils. **(B)** Representative histograms of nuclear DNA fragmentation. K shows the degree of hypodiploid (apoptotic) cells.

Neutrophils make up the main and rapidly renewing part of blood leukocytes. It is generally agreed that the physiological form of cell death in neutrophils is apoptosis (Geering and Simon, 2011). The entry of a cell into apoptosis takes a time. Neutrophils did not undergo apoptosis during 20 min adhesion (Galkina et al., 2021). To study the effect of imipramine on neutrophil apoptosis, we used standard methodological protocols, which provide for long-term incubation of cells with the test substance. In our case, neutrophils were incubated with imipramine for 4 or 18 h. We studied phosphatidylserine externalization and membrane integrity of neutrophils exposed to imipramine for 4 at 37°C using Alexa Fluor-conjugated Annexin V/propidium iodide double labeling followed by flow cytometry. Аfter 4 h incubation with 100 µM imipramine only 1.9% of cells underwent late apoptosis (Figure 4A A, region B2) and 0.2% of cells underwent necrosis (Figure 4A, region B1). Incipient apoptosis is evidenced by an increase in the number of cells with early apoptosis (Figure 4A, region B4), in which phosphatidylserine is exposed on the cell surface, but the cells retain their integrity. The effect of imipramine at a concentration of 10 μM was insignificant for early apoptosis.

To assess DNA fragmentation (Figure 4B), we incubated neutrophils with imipramine for 18 h. Neutrophils are short-lived cells. The incubation period, which lasts 18 h, seems too long for neutrophils, as a significant proportion of control cells (12.5%) die during this time (Figure 4B, control). Imipramine at concentrations of 10 or 100 μM stimulated the death of neutrophils, but the effect of imipramine was weakly concentration dependent (Figure 4B).

### Imipramine Blocks Protein Secretion by Control and LPS-Stimulated Neutrophils When Adhering to Fibronectin

We studied the effect of imipramine on the composition of protein secretion by neutrophils, which adhere to fibronectin under control conditions or in the presence of LPS. Extracellular medium samples were taken from neutrophils after 20 min adhesion to fibronectin coated substrates. Proteins were extracted with a chloroform-methanol mixture. After evaporation of the solvent, the proteins of the chloroform fraction were separated by electrophoresis, subjected to hydrolysis with trypsin directly in the gel, and identified by mass spectrometric analysis (Galkina et al., 2012). Electrophoretic gels were stained with Coomassie brilliant blue, which allows the identification of proteins by mass spectrometry. Coomassie brilliant blue stained not all, but the main secreted proteins that form a stable protein profile of neutrophil secretion, specific for each treatment (Galkina et al., 2012; Galkina et al., 2017).

**FIGURE 5:**
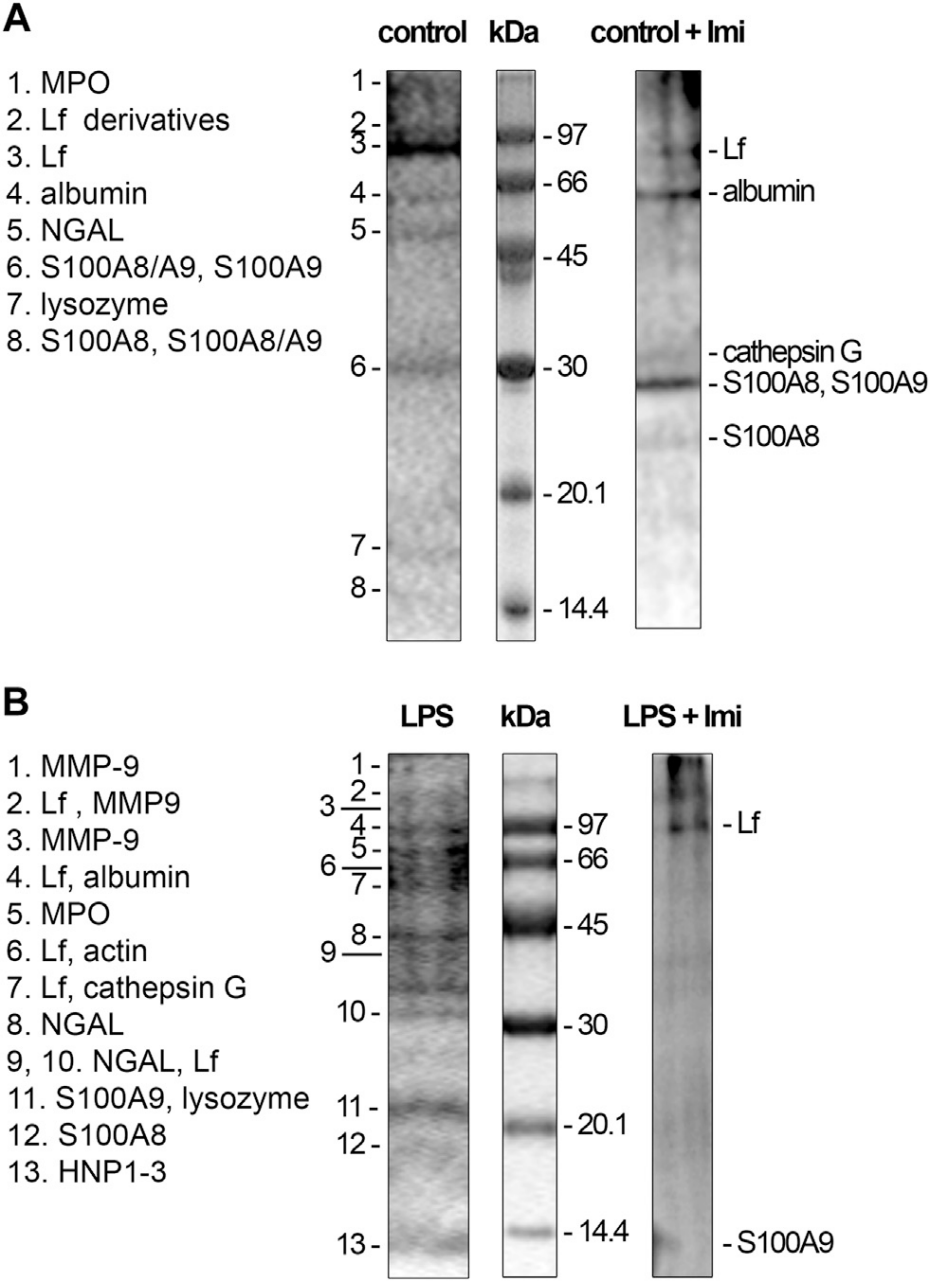
Inhibition of protein secretion of control and LPS-treated neutrophils during adhesion to fibronectin by imipramine. **(A)** Human neutrophils were attached to fibronectin-coated substrates during 25 min incubation under control conditions or in the presence of 100 μM imipramine. **(B)** Human neutrophils were attached to fibronectin-coated substrata for 25 min in the presence of 10 μg/ml LPS or 10 µg/ml LPS in combination with 100 μM imipramine. Samples of extracellular medium were collected, and proteins were extracted and subjected to separation in 15% SDS-PAGE under unreduced conditions. Gels are stained with Coomassie brilliant blue. Pictures represent typical protein profiles observed in the three independent experiments.

The protein profile of secretion of neutrophils that adhered to fibronectin under control conditions included proteins of primary (MPO) and secondary (LF, NGAL, lysozyme) granules, albumin and cytosolic S100A8 and S100A9 proteins (Figure 5A). This protein profile coincided with the protein content in the secretion of neutrophils during adhesion to fibronectin, which was previously published (Galkina et al., 2012). When neutrophils adhered to fibronectin in the presence of imipramine the protein profile of secretion did not contain NGAL and lysozyme, but was enriched with primary granule bactericide protease cathepsin G (Figure 5A, Table 1). The Mascot files obtained for all protein bands of the control + imipramine gel (Figure 5A) are presented in the supplementary file. The Mascot files contain all information about protein identification, including accuracy.

**TABLE 1:** List of proteins secreted by control and LPS-stimulated neutrophils in adherence to fibronectin in the presence of imipramine. Neutrophils were attached to fibronectin for 25 min in the presence of 100 μM imipramine or in the presence of 10 μg/ml LPS plus 100 μM imipramine. Proteins were separated by SDS-PAGE and identified by mass spectrometric analysis. Proteins identified in three analogous experiments were included in the list.

Treatment	Protein name		Peptides matched/total	Coverage %	MOWSE score
Imipramine	TRFL_HUMAN	LF	11/21	19	96
	ALBU_HUMAN	Albumin	7/12	12	69
	CATG_HUMAN	Cathepsin G	7/7	23	119
	S10A9_HUMAN	S100-A9	8/25	65	109
	S10A8_HUMAN	S100-A8	7/25	52	89
LPS + imipramine	TRFL_HUMAN	LF	24/75	31	150
	S10A9_HUMAN	S100A9	8/47	53	82

The secretion of neutrophils, which attach to fibronectin in the presence of LPS, contained the same proteins as the control cells, plus the tertiary granule component MMP-9, the primary granule components cathepsin G and defensins, and the cytoplasmic protein actin (Figure 5B). This profile coincided with the protein profile of neutrophil secretion during adhesion to fibronectin in the presence of LPS previously published (Galkina et al., 2017). The data showed that LPS stimulated the release of MMP-9, which play an important role in neutrophil adhesion and migration through its ability to modulate the extracellular matrix and remove basement membrane barriers (Dejonckheere et al., 2011). MMP-9 was secreted in parallel with NGAL, which forms complexes with MMP-9 after these components enter the extracellular environment. The NGAL complex supports allosteric activation of MMP-9 and/or protects MMP-9 from degradation by tissue metalloproteinase inhibitors, thereby maintaining enzyme activity (Tschesche et al., 2001; Yan et al., 2001).

LPS also stimulated the secretion of cathepsin G and HNP 1-3 (human neutrophil peptides 1-3 or defensins), aggressive primary granule bactericides that can initiate inflammation in surrounding tissues. In the extracellular environment, cathepsin G can interact with LF, which can serve as an allosteric enhancer of its proteolytic activity (Eipper et al., 2016).

Imipramine inhibited protein secretion by neutrophils treated with LPS upon adhesion to fibronectin. Only two major proteins, LF and S100A9, were identified in the extracellular environment of neutrophils, which attached to fibronectin in the presence of LPS and imipramine (Figure 5B, Table 1). The Mascot files obtained for all protein bands of the LPS + imipramine gel (Figure 5B) are presented in the supplementary file. Imipramine excluded MMP-9 and NGAL, cathepsin G, defensins and other proteins from neutrophil secretion during adhesion to fibronectin in the presence of LPS.

### Imipramine Selectively Inhibits the Secretion of the Free Amino Acid Hydroxylysine by Neutrophils During Adhesion Under Control Conditions or in the Presence of LPS

**FIGURE 6:**
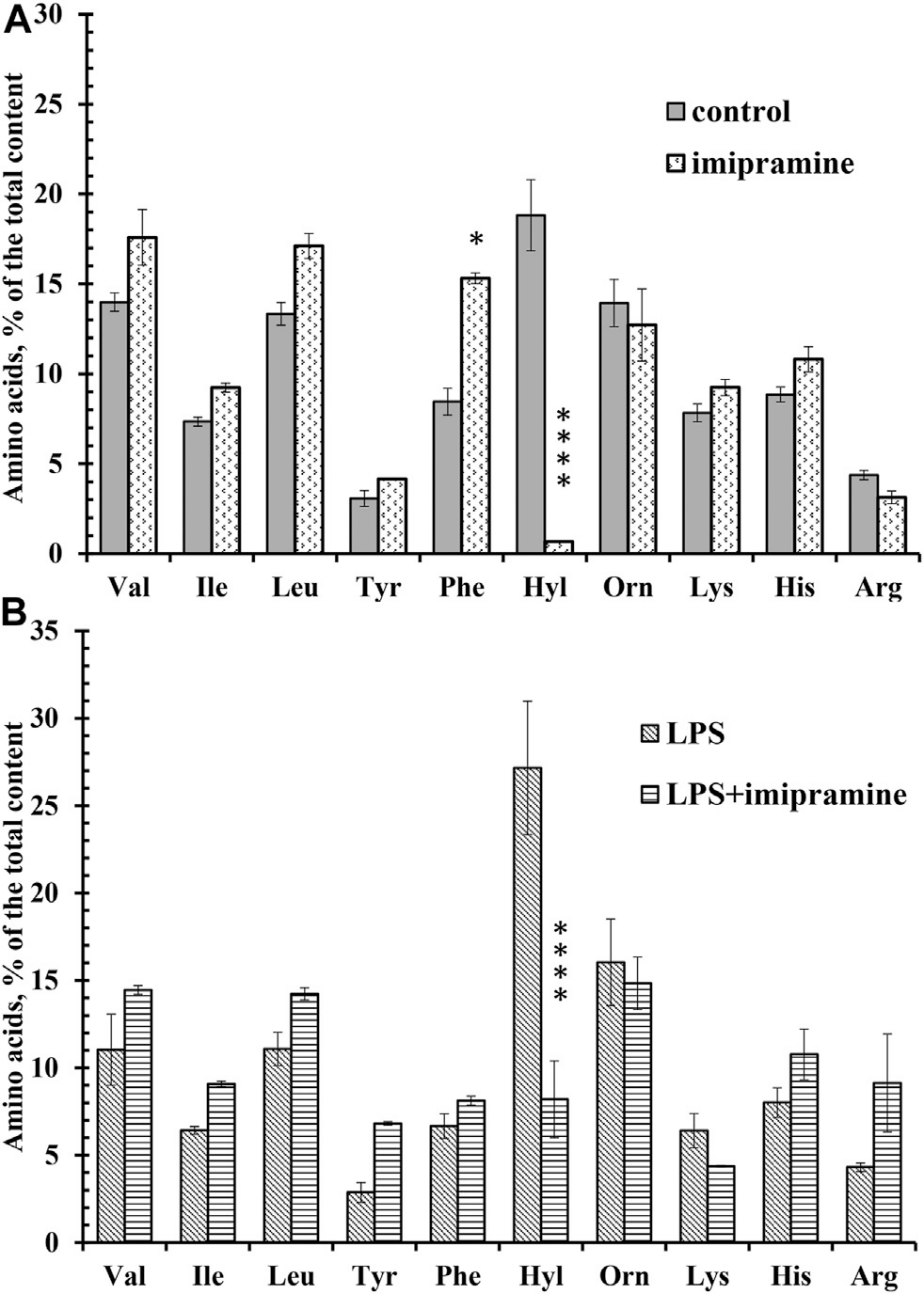
Imipramine changed the composition of free amino acid secretion by neutrophils during adhesion. **(A)** Human neutrophils were attached to fibronectin-coated substrata for 25 min under control conditions or in the presence of 100 μM imipramine. The amount of amino acid is represented as a percentage of the total content of the detected free amino acids (mean ± SEM). Amino acid profiles were obtained by summing the results of three independent experiments. ****- significant differences when compared to the value for the same amino acid in the control cells (*p* < 0.0001) as indicated by a two-way ANOVA with a Tukey’s multiple comparisons test. **(B)** Human neutrophils were attached to fibronectin-coated substrata for 25 min in the presence of 10 μg/ml LPS or in the presence of 10 μg/ml LPS and 100 μM imipramine. The amount of amino acid is represented as a percentage of the total content of the detected free amino acids (mean ± SEM). Amino acid profiles were obtained by summing the results of three independent experiments. ****- significant differences when compared to the value for the same amino acid in the presence of LPS only (*p* < 0.0001) as indicated by a two-way ANOVA with a Tukey’s multiple comparisons test.

Using amino acid analysis, we compared the free amino acid composition of the extracellular environment of neutrophils taken after adhesion of cells to fibronectin under control conditions or in the presence of imipramine. Proteins from the extracellular medium were removed by precipitation with sulfosalicylic acid and subsequent centrifugation. To make sure that the presence of free amino acids in the extracellular medium is not a consequence of cell destruction, we stained neutrophils after collecting the extracellular medium with trypan blue. The percentage of stained (dead) cells was less than 1% for control or imipramine-treated cells. In the previous studies, the amino acid composition of the extracellular medium sampled after adhesion of neutrophils to fibronectin was determined. The free amino acid profile of neutrophil secretion includes branched-chain (valine, isoleucine, and leucine), aromatic (tyrosine and phenylalanine), and positively charged amino acids (hydroxylysine, ornithine, lysine, histidine, and arginine) (Galkina et al., 2019). A similar profile of free amino acids in the extracellular medium of control neutrophils was observed in this work, which indicates that the amino acid composition of the secretion is a characteristic and stable property of neutrophils (Figure 6A). Further studies showed that the secretion of only one amino acid depends on cell adhesion—hydroxylysine, and the secretion of all other amino acids is a characteristic property of neutrophils and does not depend on cell adhesion. Almost complete and selective blocking of hydroxylysine secretion occurred in experiments when neutrophils were incubated not over fibronectin, but over a non-sticky substrate (Galkina et al., 2019). In this work, imipramine selectively and almost completely suppressed the release of hydroxylysine and caused a statistically significant increase in the content of phenylalanine (Figure 6A). Imipramine had practically no effect on the release of other amino acids by neutrophils.

The secretion profile of neutrophils that adhered to the substrate in the presence of LPS did not differ significantly from that of control cells, but the release of hydroxylysine was significantly increased compared to control cells (Figure 6B). In the presence of LPS, the percentage of hydroxylysine from the total content of detected amino acids was 27 ± 4%, but 19 ± 2% (mean ± standard error of the mean) under control conditions (*n* = 5; *p* < 0.001). Imipramine caused a statistically significant decrease in the release of hydroxylysine in the presence of LPS, but did not significantly affect the release of other amino acids by neutrophils treated with LPS (Figure 6B).

## Discussion

A growing body of research demonstrates a strong link between oxidative stress and MDD and related diseases. Repetitive stress can contribute to depressive behavior through the activation of NADPH oxidase and the resulting metabolic oxidative stress. Moreover, inhibition of NADPH oxidase has an antidepressant effect, which was found in experiments on mice (Seo et al., 2012). Neutrophils can contribute significantly to oxidative stress. The production of ROS by the NADPH complex is the characteristic ability of neutrophils to fight microbes (Segal, 2005; Winterbourn and Kettle, 2013). However, our data showed that the anti-inflammatory effect of imipramine is not associated with inhibition of ROS production by neutrophils. In the concentration range from 10 to 100 μM, imipramine stimulated but did not inhibit the production of ROS in neutrophils during adhesion to fibronectin (Figure 3). Similar results were obtained in experiments on rats. Liver oxidative stress caused by chronic mild stress was not prevented, but increased by long-term imipramine therapy (Duda et al., 2016).

At the same time, imipramine induced apoptosis in neutrophils (Figure 4). It is the accumulation of intracellular ROS that often leads to apoptosis of neutrophils (Geering and Simon, 2011; Golenkina et al., 2019). Our data demonstrating the ability of imipramine to induce apoptosis of neutrophils (Figure 4) are consistent with previously published studies demonstrating that imipramine can induce apoptosis by activating of caspase-3 and the production of ROS in YL-60 cells of acute myeloid leukemia (Xia et al., 1999a; Xia et al., 1999b). Imipramine successfully triggered apoptosis and suppressed the ability of glioblastoma cells to invade/migrate (Hsu et al., 2020).

The anti-inflammatory effect of imipramine may be related to its ability to suppress the secretion of neutrophils, which supports the processes of invasion and adhesion. The protein secretion profile of control neutrophils that attach to fibronectin in the presence of imipramine did not contain NGAL (Figure 5A, Table 1). NGAL is a 25 kDa glycoprotein of the lipocalin superfamily (Schmidt-Ott et al., 2007) originally purified from human neutrophils (Kjeldsen et al., 2000), which mediates an innate immune response to bacterial infection by sequestrating iron (Flo et al., 2004). Elevated plasma levels of this protein are associated with both cardiovascular disease and depression (Naudé et al., 2014; Gouweleeuw et al., 2015). Increased circulating NGAL levels are significantly associated with depression in the elderly and in late-life depression (Naudé et al., 2013). NGAL was demonstrated to have chemotactic properties, as neutrophils were shown to migrate along increasing concentrations of NGAL. Neutrophils of NGAL−/− mice showed a decreased neutrophil adherence and a reduced chemotactic activity (Schroll et al., 2012). In this regard, the suppression of NGAL secretion by imipramine can reduce recruitment of neutrophils to the nervous tissues. The concentration of NGAL in the CNS is very low under physiological conditions, but it is strongly induced by the administration of peripheral lipopolysaccharides (LPS). These data mean that peripheral inflammation leads to an upregulation of NGAL in the brain (Marques et al., 2008). In our experiments, LPS stimulated the secretion of a number of proteins by neutrophils (Figure 5B). Imipramine inhibited the secretion of many of these, including MMP-9, NGAL, and cathepsin G (Figure 5B, Table 1). MMP-9 plays a key role in neutrophil migration. Inhibition of MMP activity impairs neutrophil migration into the airspace of the lungs and reduces acute lung injury caused by *Streptococcus pneumoniae*, LPS, or lung injury associated with pancreatitis (Fujita et al., 2007; Sochor et al., 2009; Moon et al., 2012). The degradation of tight junction proteins by MMP-2 and MMP-9 secreted by leukemic cells increases the permeability of blood-brain barrier, which promotes invasion of leukemic cells into the central nervous system. (Feng et al., 2011). Inhibition of the secretion of MMP-9 and NGAL that form a complex supporting the enzymatic activity of MMP-9, by imipramine can significantly reduce the ability of neutrophils to invade.

We also hypothesize that blocking the release of hydroxylysine by control and LPS-stimulated neutrophils by imipramine may also interfere with the invasion of neutrophils into tissue (Figures 6A,B). The formation of hydroxylysine appears to be the result of the activation of lysyl hydroxylase during adhesion. The increased expression of this enzyme correlates with the increased ability of tumor cells to form metastases (Schiarea et al., 2010; Baek et al., 2018; Tsai et al., 2018; Verano-Braga et al., 2018; Baek et al., 2019). PLOD3 knockdown suppresses the malignant progression of renal carcinoma (Xie et al., 2020). PLOD2 knockdown inhibited glioma cell proliferation, migration and invasion (Song et al., 2017). PLOD2 can act as a direct regulator of cancer invasion/metastasis through specific interactions with integrin β1, a member of the integrin family of adhesion receptors. It has been shown that PLOD2 is able to activate β1 integrin expressed in the head and neck squamous cell carcinomas (Ueki et al., 2020; Saito et al., 2021). Inhibition of the release of hydroxylysine by imipramine in neutrophils upon adhesion to fibronectin may reflect inhibition of lysyl hydroxylase activity required for neutrophil invasion and adhesion. Our previous data have shown that adhesion-induced LH activation in neutrophils occurs in close interaction with MMPs, the PI3K/Akt pathway and intact actin cytoskeleton (Galkina et al., 2021).

The ability of neutrophils to migrate and penetrate into the tissues of the body has similarities with the ability of tumor cells to form metastases. The anti-invasive and anti-proliferative activity of imipramine has been demonstrated on various cancer cells such as small cell lung cancer, neuroendocrine pancreatic cancer, and prostate cancer cells (Jahchan et al., 2013; Barlaz Us et al., 2019). Imipramine (50–100 μM) attenuates cell migration and invasion of metastatic castration-resistant PC-3 prostate cancer cells or colorectalcancer cells (Alburquerque-González et al., 2020; Lim et al., 2020). Recently, imipramine blue, an organic derivative of the antidepressant drug imipramine, has been shown to effectively repress invasion of glioma or head and neck squamous carcinoma cells (Munson et al., 2012; Yang et al., 2016).

The results of our work suggest that imipramine can suppress the recruitment of neutrophils in the tissues of the nervous system by inhibiting cell adhesion and concomitant secretion. At the same time, imipramine demonstrated a pro-oxidant effect by stimulating the production of ROS by neutrophils. Treatment of depression and related diseases with imipramine is carried out for a long time therefore, the reduction of the toxic effect of the drug is of great importance. We suggest that the combined use of imipramine, which blocks the invasion of neutrophils in tissue, and antioxidants, which block the pro-oxidative effect of imipramine, can significantly increase the effectiveness of this antidepressant. This kind of work is being done. The synergistic effect of the combined use of classical antioxidants α-tocopherol or N-acetylcysteine with imipramine was obtained in the treatment of depressive-like behavior in experimental animals (Costa-Campos et al., 2013; Manosso et al., 2013).

## Data Availability

The original contributions presented in the study are included in the article/Supplementary Material, further inquiries can be directed to the corresponding author.

## References

[B1] Alburquerque-González B. Bernabé-García M. Montoro-García S. Bernabé-García Á. Rodrigues P. C. Ruiz Sanz J. (2020). New Role of the Antidepressant Imipramine as a Fascin1 Inhibitor in Colorectal Cancer Cells. Exp. Mol. Med. 52 (2), 281–292. 10.1038/s12276-020-0389-x 32080340 PMC7062870

[B2] Alexander C. Rietschel E. T. (2001). Invited Review: Bacterial Lipopolysaccharides and Innate Immunity. J. Endotoxin Res. 7 (3), 167–202. 10.1177/09680519010070030101 11581570

[B3] Baek J. H. Yun H. S. Kwon G. T. Kim J. Y. Lee C. W. Song J. Y. (2018). PLOD3 Promotes Lung Metastasis via Regulation of STAT3. Cel Death Dis. 9 (12), 1138. 10.1038/s41419-018-1186-5 PMC6237925 30442941

[B4] Baek J. H. Yun H. S. Kwon G. T. Lee J. Kim J. Y. Jo Y. (2019). PLOD3 Suppression Exerts an Anti-tumor Effect on Human Lung Cancer Cells by Modulating the PKC-delta Signaling Pathway. Cel Death Dis. 10 (3), 156. 10.1038/s41419-019-1405-8 PMC6377650 30770789

[B5] Barlaz Us S. Sogut F. Yildirim M. Yetkin D. Yalin S. Yilmaz S. N. (2019). Effect of Imipramine on Radiosensitivity of Prostate Cancer: An *In Vitro* Study. Cancer Invest. 37 (9), 489–500. 10.1080/07357907.2019.1662434 31496302

[B6] Brinn A. Stone J. (2020). Neutrophil-lymphocyte Ratio across Psychiatric Diagnoses: a Cross-Sectional Study Using Electronic Health Records. BMJ open 10 (7), e036859. 10.1136/bmjopen-2020-036859 PMC7371128 32690528

[B7] Carlessi A. S. Borba L. A. Zugno A. I. Quevedo J. Reus G. Z. (2019). Gut Microbiota-Brain axis in Depression: The Role of Neuroinflammation. Eur. J. Neurosci. 53 (1), 222–223. 10.1111/ejn.14631 31785168

[B8] Chen Y. Guo H. Terajima M. Banerjee P. Liu X. Yu J. (2016). Lysyl Hydroxylase 2 Is Secreted by Tumor Cells and Can Modify Collagen in the Extracellular Space. J. Biol. Chem. 291 (50), 25799–25808. 10.1074/jbc.m116.759803 27803159 PMC5207055

[B9] Costa-Campos L. Herrmann A. P. Pilz L. K. Michels M. Noetzold G. Elisabetsky E. (2013). Interactive Effects of N-Acetylcysteine and Antidepressants. Prog. Neuro-Psychopharmacology Biol. Psychiatry 44, 125–130. 10.1016/j.pnpbp.2013.02.008 23419244

[B10] Dejonckheere E. Vandenbroucke R. E. Libert C. (2011). Matrix Metalloproteinases as Drug Targets in Ischemia/reperfusion Injury. Drug Discov. Today 16 (17-18), 762–778. 10.1016/j.drudis.2011.06.009 21745586

[B11] Duda W. Curzytek K. Kubera M. Iciek M. Kowalczyk-Pachel D. Bilska-Wilkosz A. (2016). The Effect of Chronic Mild Stress and Imipramine on the Markers of Oxidative Stress and Antioxidant System in Rat Liver. Neurotox Res. 30 (2), 173–184. 10.1007/s12640-016-9614-8 26961706 PMC4947122

[B12] Eipper S. Steiner R. Lesner A. Sienczyk M. Palesch D. Halatsch M. E. (2016). Lactoferrin Is an Allosteric Enhancer of the Proteolytic Activity of Cathepsin G. PloS one 11 (3), e0151509. 10.1371/journal.pone.0151509 26986619 PMC4795699

[B13] Feng S. Cen J. Huang Y. Shen H. Yao L. Wang Y. (2011). Matrix Metalloproteinase-2 and -9 Secreted by Leukemic Cells Increase the Permeability of Blood-Brain Barrier by Disrupting Tight junction Proteins. PloS one 6 (8), e20599. 10.1371/journal.pone.0020599 21857898 PMC3157343

[B14] Flo T. H. Smith K. D. Sato S. Rodriguez D. J. Holmes M. A. Strong R. K. (2004). Lipocalin 2 Mediates an Innate Immune Response to Bacterial Infection by Sequestrating Iron. Nature 432 (7019), 917–921. 10.1038/nature03104 15531878

[B15] Fujita M. Harada E. Ikegame S. Ye Q. Ouchi H. Inoshima I. (2007). Doxycycline Attenuated Lung Injury by its Biological Effect Apart from its Antimicrobial Function. Pulm. Pharmacol. Ther. 20 (6), 669–675. 10.1016/j.pupt.2006.08.006 17045828

[B16] Galkina S. I. Fedorova N. V. Ksenofontov A. L. Serebryakova M. V. Golenkina E. A. Stadnichuk V. I. (2021). Neutrophil Adhesion and the Release of the Free Amino Acid Hydroxylysine. Cells 10 (3). 10.3390/cells10030563 PMC7999338 33807594

[B17] Galkina S. I. Fedorova N. V. Serebryakova M. V. Arifulin E. A. Stadnichuk V. I. Baratova L. A. (2017). Mold Alkaloid Cytochalasin D Modifies the Morphology and Secretion of fMLP-, LPS-, or PMA-Stimulated Neutrophils upon Adhesion to Fibronectin. Mediators Inflamm. 2017, 4308684. 10.1155/2017/4308684 28740333 PMC5504967

[B18] Galkina S. I. Fedorova N. V. Ksenofontov A. L. Stadnichuk V. I. Baratova L. A. Sud’Ina G. F. (2019). Neutrophils as a Source of Branched-Chain, Aromatic and Positively Charged Free Amino Acids. Cell Adhes. Migration 13 (1), 98–105. 10.1080/19336918.2018.1540903 PMC6527394 30359173

[B19] Galkina S. I. Fedorova N. V. Serebryakova M. V. Romanova J. M. Golyshev S. A. Stadnichuk V. I. (2012). Proteome Analysis Identified Human Neutrophil Membrane Tubulovesicular Extensions (Cytonemes, Membrane Tethers) as Bactericide Trafficking. Biochim. Biophys. Acta (Bba) - Gen. Subjects 1820 (11), 1705–1714. 10.1016/j.bbagen.2012.06.016 22766193

[B20] Geering B. Simon H.-U. (2011). Peculiarities of Cell Death Mechanisms in Neutrophils. Cell Death Differ 18 (9), 1457–1469. 10.1038/cdd.2011.75 21637292 PMC3178425

[B21] Ginis I. Zaner K. Wang J. S. Pavlotsky N. Tauber A. I. (1992). Comparison of Actin Changes and Calcium Metabolism in Plastic- and Fibronectin-Adherent Human Neutrophils. J. Immunol. 149 (4), 1388–1394. 1500723

[B22] Golenkina E. A. Viryasova G. M. Galkina S. I. Arifulin E. A. Gaponova T. V. Romanova Y. M. (2019). Synthetic CpG Oligonucleotides as Potential Modulators of Neutrophil Survival in PAMP-Associated Inhibition of Apoptosis. J. Leukoc. Biol. 106 (1), 45–55. 10.1002/jlb.3mia1118-435r 30835888

[B23] Gong X. Lu Z. Feng X. Yu C. Xue M. Yu L. (2020). Elevated Neutrophil-To-Lymphocyte Ratio Predicts Depression after Intracerebral Hemorrhage. Ndt Vol. 16, 2153–2159. 10.2147/ndt.s269210 PMC7518785 33061386

[B24] Gouweleeuw L. Naudé P. J. W. Rots M. DeJongste M. J. L. Eisel U. L. M. Schoemaker R. G. (2015). The Role of Neutrophil Gelatinase Associated Lipocalin (NGAL) as Biological Constituent Linking Depression and Cardiovascular Disease. Brain Behav. Immun. 46, 23–32. 10.1016/j.bbi.2014.12.026 25576802

[B25] Hashioka S. (2011). Antidepressants and Neuroinflammation: Can Antidepressants Calm Glial Rage Down?. Mrmc 11 (7), 555–564. 10.2174/138955711795906888 21699486

[B26] Hsu F.-T. Chiang I. T. Wang W. S. (2020). Induction of Apoptosis through Extrinsic/intrinsic Pathways and Suppression of ERK/NF‐κB Signalling Participate in Anti‐glioblastoma of Imipramine. J. Cel Mol Med 24 (7), 3982–4000. 10.1111/jcmm.15022 PMC7171418 32149465

[B27] Ishikawa Y. Kitaoka S. Kawano Y. Ishii S. Suzuki T. Wakahashi K. (2020). Repeated Social Defeat Stress Induces Neutrophil Mobilization in Mice: Maintenance after Cessation of Stress and Strain-dependent Difference in Response. Br. J. Pharmacol. 178 (4), 827–844. 10.1111/bph.15203 32678951

[B28] Jahchan N. S. Dudley J. T. Mazur P. K. Flores N. Yang D. Palmerton A. (2013). A Drug Repositioning Approach Identifies Tricyclic Antidepressants as Inhibitors of Small Cell Lung Cancer and Other Neuroendocrine Tumors. Cancer Discov. 3 (12), 1364–1377. 10.1158/2159-8290.cd-13-0183 24078773 PMC3864571

[B29] Kanashiro A. Hiroki C. H. da Fonseca D. M. Birbrair A. Ferreira R. G. Bassi G. S. (2020). The Role of Neutrophils in Neuro-Immune Modulation. Pharmacol. Res. 151, 104580. 10.1016/j.phrs.2019.104580 31786317 PMC7023896

[B30] Kjeldsen L. Cowland J. B. Borregaard N. (2000). Human Neutrophil Gelatinase-Associated Lipocalin and Homologous Proteins in Rat and Mouse. Biochim. Biophys. Acta 1482 (1-2), 272–283. 10.1016/s0167-4838(00)00152-7 11058768

[B31] Kopschina Feltes P. Doorduin J. Klein H. C. Juárez-Orozco L. E. Dierckx R. A. Moriguchi-Jeckel C. M. (2017). Anti-inflammatory Treatment for Major Depressive Disorder: Implications for Patients with an Elevated Immune Profile and Non-responders to Standard Antidepressant Therapy. J. Psychopharmacol. 31 (9), 1149–1165. 10.1177/0269881117711708 28653857 PMC5606303

[B32] Lim E. Y. Park J. Kim Y. T. Kim M. J. (2020). Imipramine Inhibits Migration and Invasion in Metastatic Castration-Resistant Prostate Cancer PC-3 Cells via AKT-Mediated NF-kappaB Signaling Pathway. Molecules 25 (20). 10.3390/molecules25204619 PMC7587212 33050597

[B33] Lynall M.-E. Turner L. Bhatti J. Cavanagh J. de Boer P. Mondelli V. (2020). Peripheral Blood Cell-Stratified Subgroups of Inflamed Depression. Biol. Psychiatry 88 (2), 185–196. 10.1016/j.biopsych.2019.11.017 32000983

[B34] Maes M. Kubera M. Leunis J. C. (2008). The Gut-Brain Barrier in Major Depression: Intestinal Mucosal Dysfunction with an Increased Translocation of LPS from Gram Negative Enterobacteria (Leaky Gut) Plays a Role in the Inflammatory Pathophysiology of Depression. Neuro Endocrinol. Lett. 29 (1), 117–124. 18283240

[B35] Maes M. Van der Planken M. Stevens W. J. Peeters D. DeClerck L. S. Bridts C. H. (1992). Leukocytosis, Monocytosis and Neutrophilia: Hallmarks of Severe Depression. J. Psychiatr. Res. 26 (2), 125–134. 10.1016/0022-3956(92)90004-8 1613679

[B36] Manosso L. M. Neis V. B. Moretti M. Daufenbach J. F. Freitas A. E. Colla A. R. (2013). Antidepressant-like Effect of α-tocopherol in a Mouse Model of Depressive-like Behavior Induced by TNF-α. Prog. Neuro-Psychopharmacology Biol. Psychiatry 46, 48–57. 10.1016/j.pnpbp.2013.06.012 23816813

[B37] Marques F. Rodrigues A.-J. Sousa J. C. Coppola G. Geschwind D. H. Sousa N. (2008). Lipocalin 2 Is a Choroid Plexus Acute-phase Protein. J. Cereb. Blood Flow Metab. 28 (3), 450–455. 10.1038/sj.jcbfm.9600557 17895910

[B38] Mazza M. G. Lucchi S. Tringali A. G. M. Rossetti A. Botti E. R. Clerici M. (2018). Neutrophil/lymphocyte Ratio and Platelet/lymphocyte Ratio in Mood Disorders: A Meta-Analysis. Prog. Neuro-Psychopharmacology Biol. Psychiatry 84 (Pt A), 229–236. 10.1016/j.pnpbp.2018.03.012 29535038

[B39] Menegazzi R. Busetto S. Decleva E. Cramer R. Dri P. Patriarca P. (1999). Triggering of Chloride Ion Efflux from Human Neutrophils as a Novel Function of Leukocyte Beta 2 Integrins: Relationship with Spreading and Activation of the Respiratory Burst. J. Immunol. 162 (1), 423–434. 9886416

[B40] Moon A. Gil S. Gill S. E. Chen P. Matute-Bello G. (2012). Doxycycline Impairs Neutrophil Migration to the Airspaces of the Lung in Mice Exposed to Intratracheal Lipopolysaccharide. J. Inflamm. 9 (1), 31. 10.1186/1476-9255-9-31 PMC3464710 22943365

[B41] Munson J. M. Fried L. Rowson S. A. Bonner M. Y. Karumbaiah L. Diaz B. (2012). Anti-invasive Adjuvant Therapy with Imipramine Blue Enhances Chemotherapeutic Efficacy against Glioma. Sci. translational Med. 4 (127), 127ra36. 10.1126/scitranslmed.3003016 22461640

[B42] Naudé P. J. W. Eisel U. L. M. Comijs H. C. Groenewold N. A. De Deyn P. P. Bosker F. J. (2013). Neutrophil Gelatinase-Associated Lipocalin: a Novel Inflammatory Marker Associated with Late-Life Depression. J. psychosomatic Res. 75 (5), 444–450. 10.1016/j.jpsychores.2013.08.023 24182633

[B43] Naudé P. J. W. Mommersteeg P. M. C. Zijlstra W. P. Gouweleeuw L. Kupper N. Eisel U. L. M. (2014). Neutrophil Gelatinase-Associated Lipocalin and Depression in Patients with Chronic Heart Failure. Brain Behav. Immun. 38, 59–65. 10.1016/j.bbi.2013.12.023 24407045

[B44] Neumann J. Riek-Burchardt M. Herz J. Doeppner T. R. König R. Hütten H. (2015). Very-late-antigen-4 (VLA-4)-Mediated Brain Invasion by Neutrophils Leads to Interactions with Microglia, Increased Ischemic Injury and Impaired Behavior in Experimental Stroke. Acta Neuropathol. 129 (2), 259–277. 10.1007/s00401-014-1355-2 25391494

[B45] Ngo T. T. Lenhoff H. M. (1980). A Sensitive and Versatile Chromogenic Assay for Peroxidase and Peroxidase-Coupled Reactions. Anal. Biochem. 105 (2), 389–397. 10.1016/0003-2697(80)90475-3 7457843

[B46] Obuchowicz E. Bielecka A. M. Paul-Samojedny M. Pudełko A. Kowalski J. (2014). Imipramine and Fluoxetine Inhibit LPS-Induced Activation and Affect Morphology of Microglial Cells in the Rat Glial Culture. Pharmacol. Rep. 66 (1), 34–43. 10.1016/j.pharep.2013.08.002 24905304

[B47] Patel N. (2009). Targeting Leukostasis for the Treatment of Early Diabetic Retinopathy. Chddt 9 (3), 222–229. 10.2174/187152909789007052 19619127

[B48] Prinz M. Priller J. (2017). The Role of Peripheral Immune Cells in the CNS in Steady State and Disease. Nat. Neurosci. 20 (2), 136–144. 10.1038/nn.4475 28092660

[B49] Risteli M. Ruotsalainen H. Salo A. M. Sormunen R. Sipilä L. Baker N. L. (2009). Reduction of Lysyl Hydroxylase 3 Causes Deleterious Changes in the Deposition and Organization of Extracellular Matrix. J. Biol. Chem. 284 (41), 28204–28211. 10.1074/jbc.m109.038190 19696018 PMC2788872

[B50] Ritzel R. M. Lai Y.-J. Crapser J. D. Patel A. R. Schrecengost A. Grenier J. M. (2018). Aging Alters the Immunological Response to Ischemic Stroke. Acta Neuropathol. 136 (1), 89–110. 10.1007/s00401-018-1859-2 29752550 PMC6015099

[B51] Rudzki L. Szulc A. (2018). Immune Gate" of Psychopathology-The Role of Gut Derived Immune Activation in Major Psychiatric Disorders. Front. Psychiatry 9, 205. 10.3389/fpsyt.2018.00205 29896124 PMC5987016

[B52] Ruhnau J. Schulze J. Dressel A. Vogelgesang A. (2017). Thrombosis, Neuroinflammation, and Poststroke Infection: The Multifaceted Role of Neutrophils in Stroke. J. Immunol. Res. 2017, 5140679. 10.1155/2017/5140679 28331857 PMC5346374

[B53] Saito K. Mitsui A. Sumardika I. W. Yokoyama Y. Sakaguchi M. Kondo E. (2021). PLOD2-driven IL-6/STAT3 Signaling Promotes the Invasion and Metastasis of Oral Squamous Cell Carcinoma via Activation of Integrin Beta1. Int. J. Oncol. 58 (6). 10.3892/ijo.2021.5209 PMC8057293 33887877

[B54] Salo A. M. Wang C. Sipilä L. Sormunen R. Vapola M. Kervinen P. (2006). Lysyl Hydroxylase 3 (LH3) Modifies Proteins in the Extracellular Space, a Novel Mechanism for Matrix Remodeling. J. Cel. Physiol. 207 (3), 644–653. 10.1002/jcp.20596 16447251

[B55] Schiarea S. Solinas G. Allavena P. Scigliuolo G. M. Bagnati R. Fanelli R. (2010). Secretome Analysis of Multiple Pancreatic Cancer Cell Lines Reveals Perturbations of Key Functional Networks. J. Proteome Res. 9 (9), 4376–4392. 10.1021/pr1001109 20687567

[B56] Schmidt-Ott K. M. Mori K. Li J. Y. Kalandadze A. Cohen D. J. Devarajan P. (2007). Dual Action of Neutrophil Gelatinase-Associated Lipocalin. Jasn 18 (2), 407–413. 10.1681/asn.2006080882 17229907

[B57] Schofield Z. V. Woodruff T. M. Halai R. Wu M. C.-L. Cooper M. A. (2013). Neutrophils-A Key Component of Ischemia-Reperfusion Injury. Shock 40 (6), 463–470. 10.1097/shk.0000000000000044 24088997

[B58] Schroll A. Eller K. Feistritzer C. Nairz M. Sonnweber T. Moser P. A. (2012). Lipocalin-2 Ameliorates Granulocyte Functionality. Eur. J. Immunol. 42 (12), 3346–3357. 10.1002/eji.201142351 22965758

[B59] Segal A. W. (2005). How Neutrophils Kill Microbes. Annu. Rev. Immunol. 23, 197–223. 10.1146/annurev.immunol.23.021704.115653 15771570 PMC2092448

[B60] Seo J.-S. Park J.-Y. Choi J. Kim T.-K. Shin J.-H. Lee J.-K. (2012). NADPH Oxidase Mediates Depressive Behavior Induced by Chronic Stress in Mice. J. Neurosci. 32 (28), 9690–9699. 10.1523/jneurosci.0794-12.2012 22787054 PMC6622255

[B61] Sochor M. Richter S. Schmidt A. Hempel S. Hopt U. T. Keck T. (2009). Inhibition of Matrix Metalloproteinase-9 with Doxycycline Reduces Pancreatitis-Associated Lung Injury. Digestion 80 (2), 65–73. 10.1159/000212080 19494493

[B62] Song Y. Zheng S. Wang J. Long H. Fang L. Wang G. (2017). Hypoxia-induced PLOD2 Promotes Proliferation, Migration and Invasion via PI3K/Akt Signaling in Glioma. Oncotarget 8 (26), 41947–41962. 10.18632/oncotarget.16710 28410212 PMC5522040

[B63] Sud'ina G. F. Brock T. G. Pushkareva M. A. Galkina S. I. Turutin D. V. Peters-Golden M. (2001). Sulphatides Trigger Polymorphonuclear Granulocyte Spreading on Collagen-Coated Surfaces and Inhibit Subsequent Activation of 5-lipoxygenase. Biochem. J. 359 (Pt 3), 621–629. 10.1042/bj3590621 11672437 PMC1222184

[B64] Tsai C.-K. Huang L.-C. Tsai W.-C. Huang S.-M. Lee J.-T. Hueng D.-Y. (2018). Overexpression of PLOD3 Promotes Tumor Progression and Poor Prognosis in Gliomas. Oncotarget 9 (21), 15705–15720. 10.18632/oncotarget.24594 29644003 PMC5884658

[B65] Tschesche H. Zölzer V. Triebel S. Bartsch S. (2001). The Human Neutrophil Lipocalin Supports the Allosteric Activation of Matrix Metalloproteinases. Eur. J. Biochem./FEBS 268 (7), 1918–1928. 10.1046/j.1432-1327.2001.02066.x 11277914

[B66] Ueki Y. Saito K. Iioka H. Sakamoto I. Kanda Y. Sakaguchi M. (2020). PLOD2 Is Essential to Functional Activation of Integrinβ1 for Invasion/Metastasis in Head and Neck Squamous Cell Carcinomas. iScience 23 (2), 100850. 10.1016/j.isci.2020.100850 PMC6997870 32058962

[B67] Umanskiy K. Robinson C. Cave C. Williams M. A. Lentsch A. B. Cuschieri J. (2003). NADPH Oxidase Activation in Fibronectin Adherent Human Neutrophils: A Potential Role forβ1 Integrin Ligation. Surgery 134 (2), 378–383. 10.1067/msy.2003.253 12947344

[B68] Verano-Braga T. Gorshkov V. Munthe S. Sørensen M. D. Kristensen B. W. Kjeldsen F. (2018). SuperQuant-assisted Comparative Proteome Analysis of Glioblastoma Subpopulations Allows for Identification of Potential Novel Therapeutic Targets and Cell Markers. Oncotarget 9 (10), 9400–9414. 10.18632/oncotarget.24321 29507698 PMC5823648

[B69] Wang C. Ristiluoma M.-M. Salo A. M. Eskelinen S. Myllylä R. (2012). Lysyl Hydroxylase 3 Is Secreted from Cells by Two Pathways. J. Cel. Physiol. 227 (2), 668–675. 10.1002/jcp.22774 21465473

[B70] Wang J.-S. Pavlotsky N. Tauber A. I. Zaner K. S. (1993). Assembly Dynamics of Actin in Adherent Human Neutrophils. Cell Motil. Cytoskeleton 26 (4), 340–348. 10.1002/cm.970260408 8299148

[B71] Wille S. M. R. Cooreman S. G. Neels H. M. Lambert W. E. E. (2008). Relevant Issues in the Monitoring and the Toxicology of Antidepressants. Crit. Rev. Clin. Lab. Sci. 45 (1), 25–89. 10.1080/10408360701713112 18293180

[B72] Winterbourn C. C. Kettle A. J. (2013). Redox Reactions and Microbial Killing in the Neutrophil Phagosome. Antioxid. Redox Signaling 18 (6), 642–660. 10.1089/ars.2012.4827 22881869

[B73] Wittenberg G. M. Greene J. Vértes P. E. Drevets W. C. Bullmore E. T. (2020). Major Depressive Disorder Is Associated with Differential Expression of Innate Immune and Neutrophil-Related Gene Networks in Peripheral Blood: A Quantitative Review of Whole-Genome Transcriptional Data from Case-Control Studies. Biol. Psychiatry 88 (8), 625–637. 10.1016/j.biopsych.2020.05.006 32653108

[B74] Xia Z. Bergstrand A. DePierre J. W. Nässberger L. (1999). The Antidepressants Imipramine, Clomipramine, and Citalopram Induce Apoptosis in Human Acute Myeloid Leukemia HL-60 Cells via Caspase-3 Activation. J. Biochem. Mol. Toxicol. 13 (6), 338–347. 10.1002/(sici)1099-0461(1999)13:6<338::aid-jbt8>3.0.co;2-7 10487422

[B75] Xia Z. Lundgren B. Bergstrand A. DePierre J. W. Nässberger L. (1999). Changes in the Generation of Reactive Oxygen Species and in Mitochondrial Membrane Potential during Apoptosis Induced by the Antidepressants Imipramine, Clomipramine, and Citalopram and the Effects on These Changes by Bcl-2 and Bcl-XL. Biochem. Pharmacol. 57 (10), 1199–1208. 10.1016/s0006-2952(99)00009-x 11230808

[B76] Xie D. Li J. Wei S. Qi P. Ji H. Su J. (2020). Knockdown of PLOD3 Suppresses the Malignant Progression of Renal Cell Carcinoma via Reducing TWIST1 Expression. Mol. Cell. probes 53, 101608. 10.1016/j.mcp.2020.101608 32585183

[B77] Yan L. Borregaard N. Kjeldsen L. Moses M. A. (2001). The High Molecular Weight Urinary Matrix Metalloproteinase (MMP) Activity Is a Complex of Gelatinase B/MMP-9 and Neutrophil Gelatinase-Associated Lipocalin (NGAL). J. Biol. Chem. 276 (40), 37258–37265. 10.1074/jbc.m106089200 11486009

[B78] Yang W.-H. Su Y.-H. Hsu W.-H. Wang C.-C. Arbiser J. L. Yang M.-H. (2016). Imipramine Blue Halts Head and Neck Cancer Invasion through Promoting F-Box and Leucine-Rich Repeat Protein 14-mediated Twist1 Degradation. Oncogene 35 (18), 2287–2298. 10.1038/onc.2015.291 26257063 PMC5929114

